# The influence of share buybacks on ill-health and health inequity: an exploratory analysis using a socio-ecological determinants of health lens

**DOI:** 10.1186/s12992-023-00905-0

**Published:** 2023-01-11

**Authors:** Benjamin Wood, Gary Sacks

**Affiliations:** Global Centre for Preventive Health and Nutrition, Deakin University, Institute for Health Transformation, 221 Burwood Highway, Burwood, VIC 3125 Australia

**Keywords:** Share buybacks, Commercial determinants of health, Corporate determinants of health, Health inequity

## Abstract

**Introduction:**

Share buybacks, when a corporation buys back its own shares, are recognised as having potentially harmful impacts on society. This includes by contributing to economic inequalities, and by impeding investments with the potential to protect and promote the welfare of various stakeholders. Share buybacks, however, have received minimal analytical attention in the public health literature. This paper aimed to explore the potential influence of share buybacks on population health and health inequity using a socio-ecological determinants of health lens.

**Methods:**

We conducted a descriptive analysis of share buybacks made by corporations listed on United States (US) stock exchanges between 1982 and 2021, using quantitative data sourced from Compustat. We examined annual trends in share buyback expenditure, including comparisons to dividend, net income, capital expenditure, and research and development expenditure data. We then purposively sampled a set of corporations to provide illustrative examples of how share buybacks potentially influence key socio-ecological determinants of health. The examples were: i) three COVID-19 vaccine manufacturers; ii) five of the world’s largest fossil fuel corporations; and iii) US car manufacturer General Motors. For these, we conducted an analysis of data from Compustat, company reports and grey literature materials, focusing on key sources of profits and their allocation to share buybacks and particular investments.

**Results:**

US-listed corporations spent an estimated US$9.2 trillion in real terms on share buybacks between 2012 and 2021 (nearly 12 times more than from 1982 to 1991). The contribution of share buybacks to total shareholder ‘returns’ increased from 11% in 1982 to 55% in 2021, with expenditure on shareholder returns increasing considerably relative to capital, research and development expenditure over this period. The three examples illustrated how some corporations have prioritised the short-term financial interests of their shareholders, including via implementing large share buyback programs, over investments with considerable potential to protect and promote the public’s health.

**Conclusion:**

The potentially substantial impacts of share buybacks on health warrant increased research and policy attention. Arguably, more must be done to regulate share buybacks as part of efforts to address the corporate drivers of ill-health and inequity.

## Introduction

Since their inception, many for-profit business corporations (hereinafter corporations) have influenced and impacted on health to a considerable degree. Corporations can positively impact on health, such as by contributing to economic development, job creation, and socially-beneficial technological progress [1–3]. Many concerns, however, have been raised about the myriad ways by which some corporations negatively impact on society and the environment [4–6].

Public health researchers have long recognised ways in which corporations adversely influence health, especially with respect to those that produce and market commodities harmful to health (e.g., tobacco, pesticides, fossil fuels, ultra-processed foods) [7–12]. Large corporations active in numerous other sectors, including, *inter alia*, the pharmaceutical, healthcare, finance, food retail, and digital platform sectors, have also come under considerable public health scrutiny [13–19]. For example, while the pharmaceutical sector has been lauded for its involvement in the development and production of COVID-19 vaccines, many pharmaceutical corporations have been criticised for undermining efforts to make COVID-19 vaccines accessible and affordable for all [20]. Public health efforts to understand, monitor, and address the negative impacts of corporations on health have increasingly been understood as part of the ‘commercial determinants of health’, a term which encourages analysis of harmful corporate practices, as well as the underlying institutional and governance arrangements that facilitate such practices [8, 21].

The ways in which corporations distribute wealth and income can have a considerable influence on population health and health equity [22–24]. As we argue below, open market share buybacks (hereinafter share buybacks), referring to when a corporation buys back its own shares on the open market, are an increasingly important corporate practice in this respect. When a corporation spends money on share buybacks, it is essentially transferring this money to its shareholders by inflating share prices (as well as to company executives by influencing metrics commonly linked to executive remuneration) [25]. To date, however, limited analysis of share buybacks has occurred through a public health lens.

Along with dividend payments, share buybacks are one of two ways a corporation distributes ‘cash’ to its shareholders. Share buybacks have come to epitomise corporate short-termism, or what Mazzucato (2018) refers to as the ‘financialization of the real economy’, for a number of reasons [25, 26]. First, whereas corporations have made regular dividend payments to their shareholders for centuries, large-scale share buybacks are only a relatively recent phenomenon. Indeed, for most of the period between the early 1600s and today, dividends were generally the only way that corporations transferred cash to their shareholders [27, 28]. In comparison, in many jurisdictions, share buybacks were considered an illegal form of market manipulation, at least for most of the twentieth century [26]. Starting in the 1980s, at a time when the ‘maximising shareholder value’ ideology became increasingly legitimised and popularised, many countries chose to legalise share buybacks, including the United Kingdom (UK) in 1981, the United States (US) in 1982, Japan in 1994, and Germany in 1998 [26, 29, 30]. Moreover, dividends in moderation are generally perceived as an appropriate way of providing a yield to shareholders [31]. Share buybacks, on the other hand, serve largely to increase share prices in the short term, thereby increasing the capital gains that can be realised by shareholders, as well as executive pay [25, 28]. In recent decades, share buyback expenditure has been more volatile than dividend expenditure, with corporate executives often implementing or expanding their share buyback programs during periods of high profits in *addition* to, rather than instead of, dividend payments [32].

A range of previous studies have identified that share buybacks are an increasingly important driver of wealth and income inequalities [25, 26, 28, 32–35]. As with dividends, share buybacks directly contribute to widening wealth and income inequalities because shareholders (including the ultimate owners of related assets under management) tend to be over-represented by the wealthiest groups in mostly high-income countries [26, 28, 36–38]. In the United States (US), for example, the wealthiest 10% of households own approximately 85% of corporate equity [37], with the wealthiest 0.1% of people deriving most of their income from corporate revenues as opposed to wages [39]. Even shareholder ‘returns’ that accrue to pension funds tend to be distributed in an inequitable manner. For instance, in the United Kingdom (UK), the richest 20% of households by income own nearly 50% of pension wealth in the country [40].

Wealth and income inequalities are associated with a large range of adverse health outcomes [41–45]. At the individual and household levels, people with lower incomes have less money to spend on essential products and services, such as healthy foods, healthcare, and childcare. They are also less likely to live in safer neighbourhoods and are more likely to experience stress and mental health issues [46–49]. At a broader level, widening wealth and income inequalities within countries indirectly influence health and health equity as they are associated with, among other things, a decrease in civic engagement, a breakdown of social cohesion and trust in public institutions, and the widening of gender, racial, and intergenerational inequalities [47, 49–51]. Wealth and income inequalities between countries are a major barrier to poverty eradication and sustainable economic development [52].

Beyond directly contributing to wealth and income inequalities, share buybacks have the potential to adversely impact on health because they represent profits that corporate decision-makers have chosen not to invest in other options that may be meaningful at a societal level (e.g., innovation that provides a net benefit for society) [25, 35]. Since the 1980s, share buybacks have increasingly become a major use of corporate profits, with the practice associated with decreasing levels of long-term investment in productivity and innovation [25, 26, 28]. Thus, it appears that share buybacks have been instrumental in the operationalisation of the ‘maximising shareholder value’ ideology, which, in recent decades, has reportedly emerged to become the dominant principle underpinning corporate governance around much of the capitalist world [26, 53].

When corporate decision-makers allocate insufficient funds and resources to long-term investments, the benefits of which tend to be cumulative, collective, and uncertain, they are potentially jeopardising the welfare of many of their stakeholders (and in some cases the longevity of the corporation itself) [25, 54]. More broadly, a lack of long-term investment, especially by large and dominant corporations in productive capacities and innovation, can threaten the development and prosperity of entire economies [25, 35]. Corporate short-termism can also have a range of sector-specific consequences that influence the public’s health. For instance, decision-makers in fossil fuel corporations can jeopardise efforts to address climate and ecological breakdown – recognised by many as the greatest threat to public health [55] – when they choose to prioritise the short-term financial interests of their shareholders over transitioning towards renewable energy production [56]. As another example, decision-makers in healthcare corporations threaten equitable access to quality and affordable healthcare when they choose to prioritise the short-term financial interests of their shareholders over investing in increasing the quality and reach of existing services [57].

Given the abovementioned concerns and research gap in the public health literature, this paper aimed to explore the potential influence of share buybacks on ill-health and health inequity using a socio-ecological determinants of health lens. The goal of this study was to inform broader efforts to understand and address the ways in which corporations negatively impact on population health and health equity.

## Material and methods

To address the aims of the paper, we adopted a synthesis research design using multiple methods, involving descriptive analysis of quantitative data and document analysis of corporate reports and grey literature materials. We began with a descriptive analysis of annual share buyback expenditure, which was compared to data on dividends, net income, capital expenditure, and research and development expenditure, for corporations listed on US stock exchanges between 1982 (the year that regulation of share buybacks was relaxed in the US) and 2021. This was done to broadly explore the potential contribution of share buybacks to economic inequalities, as well as their role in impeding long-term investment. We then purposively selected three illustrative examples of how share buybacks potentially contribute to ill-health and environmental harm by representing funds that corporate decision-makers have chosen not to allocate towards meaningful investments (at a societal level). Our hypothesis was that such decisions have the potential to adversely impact on a number of key social and ecological determinants of health. For each illustrative example, we conducted an analysis of data sourced from Compustat, company reports and targeted grey literature materials. We focused on key sources of corporate profits, as well as important ‘opportunity costs’ that arise when corporations choose to allocate funds towards share buybacks. We describe these steps in further detail below.

### Conceptual framework

Rather than examining specific health outcomes, we chose to conceptualise the ways in which share buybacks impact on population health and health equity by exploring their potential influence on key social and ecological determinants of health. The social determinants of health refer to the social factors and conditions that influence health outcomes (e.g., conditions in which people are born, work and live), as well as the broader set of forces and systems that shape these factors and conditions [58]. As outlined in the introduction, share buybacks directly contribute to widening wealth and income inequalities because of the way in which corporate share ownership is distributed across society. In turn, widening wealth and income inequalities are associated with a range of adverse health outcomes. Furthermore, share buybacks are associated with a lack of long-term investment, and, in some cases, the promotion of harmful profiteering practices, that have the potential to negatively impact on health through both general and sector-specific mechanisms [25, 26, 32]. These include by jeopardising the livelihoods of workers, and by impeding access to quality essential products and services.

The ecological determinants of health recognise that health is dependent on healthy natural ecological systems and processes, and that the impacts of global ecological change on health are profound [59]. Especially in sectors such as fossil fuels, transport, and agriculture, corporate decision-makers can negatively influence key ecological determinants of health by choosing to prioritise the short-term financial interests of their shareholders, including via implementing large share buyback programs, over investing in protecting and promoting the health of natural ecological systems and processes.

### Quantitative analysis of share buyback data

Share buyback data for corporations listed on US stock exchanges and for every fiscal year between 1982 and 2021 were sourced from Compustat [60]. Given that US stock markets held nearly 60% of the total world equity market value at the beginning of 2022, we felt that US-listed corporations provided a sufficiently large sample size for the purposes of this exploratory study [61]. The focus on US-listed corporations also had the additional advantage of avoiding potential complications related to exchange rate conversions.

Our analysis focused on annual trends in share buyback expenditure. While Compustat provides data on the repurchasing of open market and preferred shares, it aggregates these into one item. As we were interested in analysing ‘open market’ share buybacks, we followed the approach outlined by Grullon and Michaely (2002) by subtracting the reductions in the value of the net number of preferred stocks outstanding from the total value of repurchases of ‘common and preferred stock’ [62]. We added share buyback data to dividend data to calculate total shareholder ‘returns’, which was then compared to:

i) Net income (a proxy for profits), a key foundation for future investment [28];
ii) Capital expenditure plus research and development expenditure, which, combined, serve as a crude proxy for actual long-term investment [63].

We presented these ratios as 5-year moving averages to facilitate the analysis of long-term trends. Where relevant and possible, nominal values were adjusted to 2021 US dollar (USD) values using the World Bank’s gross domestic product (GDP) deflator dataset [64].

### An analysis of three illustrative examples: selection of corporations and methods used

We examined three illustrative examples, selected in a purposive manner, to provide further insight into the ways in which share buybacks impact on health by representing funds that have not been allocated towards meaningful investments. These three examples were chosen because we felt that, for the purposes of this exploratory study, they illustrated a range of general and sector-specific health-related consequences of large-scale share buyback programs. In addition, the three examples demonstrated how a considerable proportion of the funds used for shareholder return programs, including share buyback programs, can often be traced back to direct and indirect financial assistance from governments. This has important normative implications for regulating share buybacks, a point to which we return in the discussion section.

The three illustrative examples were:

i) The manufacturers of the largest Coronavirus Disease 2019 (COVID-19) vaccines by revenue.
ii) The fossil fuel industry, focusing on the industry’s five largest corporations (excluding state-owned corporations).
iii) General Motors, a major US car manufacturer, focusing on its share buyback programs prior to and following the corporation’s so-called ‘Auto Bailout’ in 2009.

For the first example (COVID-19 vaccine manufacturers), we analysed financial data extracted from Compustat, as well as the annual reports (2020 to 2021) and most recent quarterly earnings reports or presentations of the manufacturers of the world’s two largest COVID-19 vaccines by global market share in 2021 [65]. These were Pfizer and BioNTech (as part of a joint venture), and Moderna. We analysed information related to share buybacks, dividends, and net income (ascertaining where possible the contribution made by COVID-19 vaccine sales to the company’s overall net income). This was complemented with a targeted grey literature search on the use of public funds and resources in the development of the respective vaccines, and on the advance purchasing agreements made by these corporations with governments during 2021.

For the second example (fossil fuel industry), we analysed quantitative data on share buybacks and dividends sourced from Compustat for the US-listed fossil fuel industry, which we considered to encompass all US-listed corporations in the oil, gas, and consumable fuels industry (Global Industry Classification Standard 101020). Given the reportedly important relationship between fossil fuel profits and crude oil prices [66], we chose to compare share buyback and dividend data to data on the price of crude oil imported into the US, which we used as a rough proxy for world crude oil prices. Oil price data were sourced from the US Energy Information Administration [67]. We then analysed the annual reports (2017 to 2021), sustainability reports (2017 to 2021), and most recent quarterly earnings reports or presentations of the world’s five largest fossil fuel corporations, excluding state-owned corporations, based on the latest revenue data at the time of data collection [68]. These corporations were: ExxonMobil, Shell, Total Energies, Chevron, and BP. Document analysis of company reports was supported with a targeted grey literature search on important sources of the industry’s profits.

For the third example (General Motors), we analysed General Motors’ annual reports from 2009 onwards (the year when the US government announced a bailout plan for the automobile corporation) [69]. Document analysis was complemented with a quantitative analysis of data sourced from Compustat, as well as from grey literature materials relating to General Motors’ share buyback programs and announcements of job cuts from 2009 onwards.

## Results

### Quantitative analysis of US-listed corporations between 1982 and 2021

**Fig. 1:**
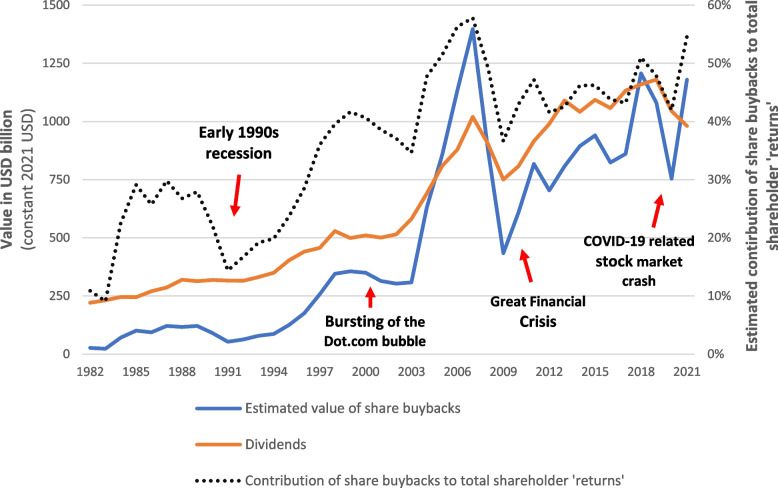
Estimated value of open market share buybacks and dividends made by US-listed corporations in USD billion, 1982–2021. Source: Compustat North America via Wharton Research Data Services. Values in constant 2021 USD. Estimated annual value of ‘open market’ share buybacks = value of common and preferred stock purchases made minus the calculated reduction in the net value of preferred stocks outstanding (when a positive value). Total shareholder returns = open market share buybacks + dividends (common) paid

Punctuated by largescale financial crises and events, estimated annual share buyback expenditure by US-listed corporations in real terms was seen to generally trend upwards between 1982 and 2021 (Fig. 1). From approximately US$27 billion in 1982 (constant 2021 USD), the annual value of share buybacks was estimated to peak at around US$1.4 trillion in 2007 – the beginning of the Great Financial Crisis (GFC) – before plummeting to nearly US$430 billion in 2009. Estimated annual share buyback expenditure was seen to increase again until 2020, a year marked by widespread recessions triggered by the COVID-19 pandemic and related events. In the ten-year period between 2012 and 2021, the total value of share buybacks was estimated at nearly US$9.2 trillion, nearly US$1.2 trillion of which occurred in 2021. Figure 1 also shows that the contribution of share buybacks to total shareholder ‘returns’ (share buybacks and dividends combined) generally increased over the period of analysis, from approximately 10% in the early 1980s to around 50% from 2018 onwards.

**Fig. 2:**
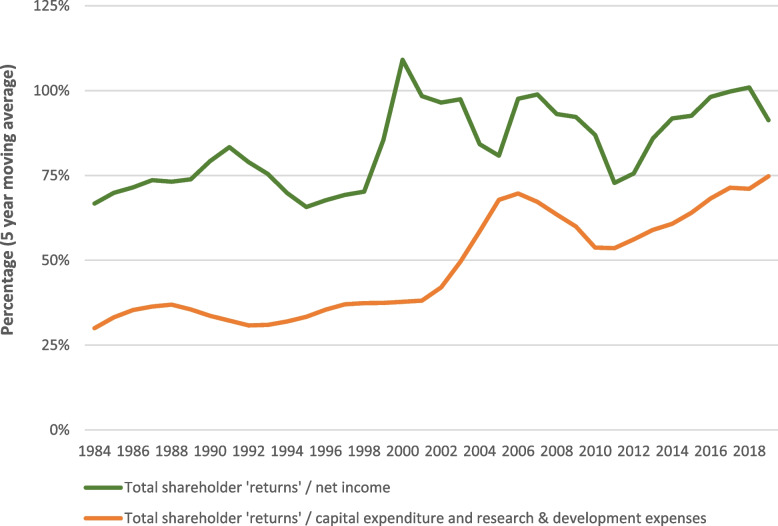
Estimated value of total shareholder returns relative to net income, as well as capital expenditure and research and development expenditure, for US-listed corporations, 1982–2021. Source: Compustat North America via Wharton Research Data Services. Estimated annual value of ‘open market’ share buybacks = value of common and preferred stock purchases made minus the calculated reduction in the net value of preferred stocks outstanding (when a positive value). Total shareholder returns = open market share buybacks + common dividends paid

Between 2012 and 2021, inclusive, aggregate spending on total shareholder returns (share buybacks and dividends) was approximately 92% of aggregate net income, compared to 71% between 1982 and 1991 (Fig. 2). The ratio of total shareholder ‘returns’ to capital, research, and development expenditure (a proxy for actual long-term investment), increased from an estimated 24% in 1982 to 88% in 2021, which was the highest annual percentage recorded over the period of analysis.

### Illustrative examples

#### COVID-19 vaccine manufacturers: Pfizer, BioNTech, and Moderna

*“Our COVID-19 vaccine deliveries and revenues exceeded our expectations. After such an extraordinary year, we would like our shareholders to participate in our strong 2021 performance through a repurchase program of BioNTech shares.”*

- BioNTech’s 2021 Annual Report [70]

The two vaccines that dominated the global COVID-19 vaccine market in 2021 and early 2022 were Comirnaty, jointly developed by Pfizer and BioNTech, and mRNA-1273, developed by Moderna [71]. The origins of these two COVID-19 vaccines can be traced backed to key publicly funded innovations and large amounts of direct government funding [72]. US federal funding, for instance, played a pivotal role in the two discoveries that were fundamental to the development of the COVID-19 vaccines commercialised by Pfizer, BioNTech, and Moderna: the discovery of a target ‘spike’ protein, and the modification of RNA (the concept that inspired Moderna’s name) [72, 73]. Additionally, the US Department of Defense, through its Defense Advanced Research Projects Agency (DARPA), made a number of important high risk investments in RNA vaccine technology, including a funding award worth US$25 million for Moderna towards developing RNA vaccines against the Zika and Chikungunya viruses in 2013 [72].

During the early stages of the COVID-19 pandemic, Pfizer, BioNTech and Moderna received a considerable amount of direct government funding to support their COVID-19 vaccine development programs. Between June and September 2020, Pfizer and BioNTech received approximately US$800 million in direct funding from the European Investment Bank, Singapore’s state investment bank, and the German Federal Ministry of Education and Research [74]. Moderna reportedly received nearly US$2.5 billion in US federal funding, as part of Operation Warp Speed, to develop its COVID-19 vaccine [75]. On top of these public funds, Pfizer, BioNTech, and Moderna were promised guaranteed revenue from governments in the form of advance purchase agreements (APAs) for their vaccines, which at the time were yet to be proven fully effective. In 2020 alone, Pfizer and BioNTech entered APAs worth a total of US$17.7 billion, and Moderna entered APAs worth US$7.4 billion [74]. Research commissioned by Public Citizen in 2021 identified that, as part of these APAs, as well as subsequent purchasing agreements of COVID-19 vaccines, governments around the world had paid between 4 and 24 times the cost of vaccine production [76]. In another report published by Oxfam, it was stated that, as of mid-2021, Pfizer, BioNTech, and Moderna had charged governments as much as US$41 billion above estimated production costs [77]. The high prices set by these corporations for COVID-19 vaccines meant that many governments of Global South countries were not able to compete with the governments of Global North countries for access to the vaccines [76, 77].

In 2021 and the early parts of 2022, Pfizer, BioNTech, and Moderna generated considerable profits from their COVID-19 vaccines. By mid-2022, all three companies had announced share buyback programs to distribute some of this so-called ‘excess capital’ to shareholders.

According to data sourced from Compustat, Pfizer reported a net income of US$22 billion in 2022, a 35% increase in real terms from 2019. This surge in net income was driven to a large extent by sales of its highly profitable COVID-19 vaccine (jointly developed with BioNTech) [78–80]. For the first three quarters of 2022, Pfizer had reported a net income of more than US$26 billion. Along with paying out around US$6.7 billion in dividends in the first three quarters of 2022 (on top of US$8.8 billion in 2021), the company bought back US$2 billion worth of its shares in the first quarter of 2022, leaving the remaining amount of the company’s authorised share buyback program at US$3.3 billion [81, 82].

For BioNTech, its COVID-19 vaccine (jointly developed with Pfizer) drove its reported net income from a loss of US$216 million in 2019 (constant 2021 USD) to a gain of more than US$11.7 billion in 2021 and US$7.0 billion for the first three quarters of 2022 [70, 83]. BioNTech, a company founded in 2008, had never previously paid out dividends or bought back its own shares prior to 2022. Between May and December 2022, the company bought back US$1 billion worth of its shares, with plans to buy back another US$500 million starting from December [84]. BioNTech also paid out a special cash dividend of around US$475 million in the first half of 2022 [84].

Moderna, like BioNTech, reported an enormous surge in net income due to its COVID-19 vaccine, which was estimated as having a 70% pre-tax profit margin in 2021 [80, 85, 86]. This enabled Moderna to turn around a net income loss of around US$550 million (constant 2021 USD) in 2019 to a net income gain of US$12.2 billion in 2021 and US$6.9 billion during the first three quarters of 2022. Prior to the final quarter of 2021, Moderna had never bought back its own common shares. For the first three quarters of 2022, the company bought back US$2.9 billion worth of its shares as part of an authorised US$6 billion share buyback program [87]. The company did not report any dividend payments during this time.

As of early 2022, over 100 countries were calling for intellectual property (IP) rules to be lifted to improve COVID-19 vaccine equity [88]. These IP rules enable pharmaceutical corporations to generate large profits from COVID-19 vaccines, and thus it is perhaps unsurprising that many pharmaceutical corporations have been and continue to be staunchly opposed to measures such as IP waivers [88]. Pfizer, as a case in point, reportedly pressured officials in South Africa to drop the nation’s IP waiver program during months of negotiations in 2021 over a contract for the supply of COVID-19 vaccines [89]. Pfizer did pledge commitment at the 2022 World Economic Forum to provide a range of vaccines and medicines at not-for-profit prices to 45 lower income countries [90]. However, some have noted that not only is this ‘too little, too late’ to improve low vaccination rates in many lower-income countries, the initiative will cost Pfizer little while helping it build a new market and its reputation [90, 91].

### The fossil fuel industry and its five largest non-state-owned corporations

*“At its meeting on February 9, 2022, the Board of Directors has defined a shareholder return policy for 2022 [... including] buybacks to share the surplus cash flow from high hydrocarbon prices.”*

- Total Energies’ 2021 Annual Report

**Fig. 3:**
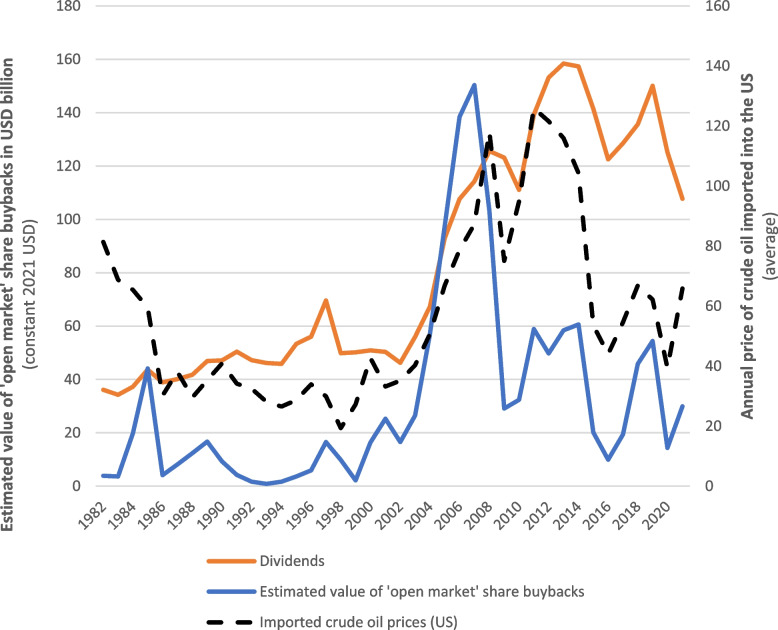
Estimated value of share buyback expenditure and dividend payments made by US-listed fossil fuel corporations versus the annual average price of crude oil imported into the US, 1982–2021. Sources: Compustat North America (via Wharton Research Data Services) and US Energy Information Administration. Values fixed to 2021 USD. Estimated annual value of ‘open market’ share repurchases = value of common and preferred stock purchases made minus the calculated reduction in the net value of preferred stocks outstanding

From the world’s first United Nations Framework Convention on Climate Change’s Conference of the Parties in 1995 up until the end of 2021, the US-listed fossil fuel industry transferred nearly US$3.8 trillion (constant 2021 USD) to its shareholders. Share buybacks accounted for more than US$1 trillion worth of these shareholder ‘returns’. Expenditure on share buybacks by US-listed fossil fuel corporations was seen to peak between 2005 and 2008, with an estimated US$435 billion (constant 2021 USD) spent on share buybacks during this period (Fig. 3). Although annual expenditure declined after the Global Financial Crisis, the industry still allocates many billions of dollars towards share buybacks every year.

Since the late 1990s, the industry’s annual expenditure on share buybacks, and to a lesser extent dividends, has somewhat correlated with crude oil prices. This is consistent with evidence that large fossil fuel corporations tend to generate large profits when global oil prices are high, as occurred during the first half of 2022 when some of the largest fossil fuel corporations generated record profits [92, 93]. Moreover, it has been recognised that the fossil fuel industry owes a considerable proportion of its profits to both its ability to externalise enormous environmental costs, as well as government subsidies [94]. For instance, a recent report published by the International Monetary Fund calculated that the environmental costs externalised by the fossil fuel industry, along with fossil fuel subsidies, reached *US$5.9 trillion* in 2020 alone.

Despite being explicit about the need for an energy transition in their sustainability reports, four of the world’s five largest fossil fuel corporations have allocated much more money towards their shareholders, including via share buybacks, than towards investing in renewable and low carbon energy programs in recent years (as reported) (Table 1). As a pertinent example, between 2002 and 2021, Exxon Mobil allocated an estimated US$318 billion (constant 2021 USD) towards share buybacks, along with US$243 billion in dividends, compared to around US$10 billion in research, development, and implementation of so-called ‘lower-emission’ energy solutions [95]. For 2022 and beyond, the same four corporations have made larger commitments to share buybacks and dividends than to investing in renewable and low carbon energy (as reported) (Table 1). After generating record profits, Shell, for instance, spent US$14.5 billion on share buybacks in the first three quarters of 2022 (on top of US$5.6 billion in dividends), and plans to spend another US$4 billion on share buybacks by the end of the year. In comparison, Shell aimed to invest US$3 billion in its renewables and ‘energy solutions’ business over the entire year [96, 97].

**Table 1:** Commitments made by the world’s five largest fossil fuel corporations (excluding state-owned corporations) to share buybacks versus investing in renewable and low carbon energy

	**Commitment to share buybacks and dividends**		**Commitment to investing in renewable and low carbon energy**	
	**In recent years**	**2022 and beyond**	**In recent years**	**2022 and beyond**
**ExxonMobil**	Between 2002 and 2021, ExxonMobil spent an estimated US$318 billion on share buybacks and US$243 billion on dividends (constant 2021 USD)	During the first three quarters of 2022, Exxon spent US$10.5 billion on share buybacks as part of a US$30 billion program to be completed by the end of 2023. During this time, the company also spent US$11.2 billion on dividends.	Between 2002 and 2021, Exxon Mobil invested more than US$10 billion in research, development, and implementation of ‘lower emission’ energy solutions (value not adjusted)	Between 2022 and end of 2027, Exxon has committed more than US$15 billion towards initiatives that will reduce emissions from their operations and advance opportunities in their Low Carbon Solutions business
**Shell**	Shell spent US$19.9 billion on share buybacks and US$47.5 billion on dividends between 2018 and 2021 (constant 2021 USD)	Shell spent US$14.5 billion on share buybacks during the first three quarters of 2022 and plans to spend another US$4 billion during the fourth quarter of 2022. The company spent US$5.6 billion on dividends during the first three quarters of 2022.	In 2020, Shell committed to investing US$2–3 billion in its ‘Renewables and Energy Solutions’ business. In 2021, Shell invested US$2.4 billion in this business.	Shell aims to invest US$3 billion in its ‘Renewables and ‘Energy Solutions’ business in 2022
**Total Energies**	Total Energies spent approximately US$10 billion on share buybacks and US$31 billion on dividends between 2017 and 2021 (constant 2021 USD)	Total Energies spent US$5 billion on share buybacks and US$5.6 billion on dividends during the first three quarters of 2022	Total Energies invested more than US$10 billion in renewable energy between 2017 and 2021 (value not adjusted)	Total Energies aims to invest more than US$60 billion in renewable power generation capacity by 2030
**Chevron**	Chevron spent approximately US$9.5 billion on share buybacks and US$39 billion on dividends between 2018 and 2021 (constant 2021 USD)	Chevron spent US$7.5 billion on share buybacks and US$8.3 billion on dividends in the first three quarters of 2022	Between 2018 and 2020, Chevron invested approximately US$1.1 billion in a range of carbon capture, utilisation, and storage projects. By July 2021, Chevron had made US$500 million worth of commitments to its low-carbon venture funds (value not adjusted).	In 2021, Chevron committed to US$10 billion in ‘lower carbon investments’ by 2028
**BP**	BP spent approximately US$6.4 billion on share buybacks and US$32.6 billion on dividends between 2017 and 2021 (constant 2021 USD)	BP spent around US$6.8 billion on share buybacks and US$3.3 billion on dividends in the first three quarters of 2022	BP allocated US$4.2 billion to low carbon investments between 2017 and 2021 (value not adjusted)	BP aims to increase low carbon investments from the US2.2 billion it made in 2021 to US$3–4 billion per year by 2025

Sources: Compustat North America (accessed via Wharton Research Data Services); company annual reports, quarterly earnings reports, sustainability reports, and share buyback related media releases (as of November 2022). Periods of analysis vary for each corporation because they present data on investment and shareholder return expenditure differently. Values adjusted to 2021 USD when possible

### General Motors and the US ‘Auto Bailout’

*“From the beginning, I made it clear that I would not put any more tax dollars on the line if it meant perpetuating the bad business decisions that had led [General Motors] to seek help [from the government] in the first place […] Understand we’re making these investments not because I want to spend the American people’s tax dollars, but because I want to protect them.”*

- Barack Obama, then-President of the US, 2009 [69]

General Motors (GM), a US-based corporation founded in 1908, was the world’s largest producer of automobiles for a 77-year period between 1931 and 2008 [98]. After facing a sustained period of poor financial performance starting in the mid-2000s, including over US$70 billion in losses in 2007 and 2008, GM filed for the largest industrial bankruptcy in US history in 2009 [98, 99]. Shortly afterwards, the US government announced a ‘bailout’ plan to provide GM with nearly US$50 billion of taxpayers’ money, as part of the Trouble Asset Relief Program created by the US Congress during the GFC [54, 100].

GM was required to restructure – re-emerging as the so-called ‘New GM’ – and a considerable part of the US government’s bailout was converted into a large equity stake in the company [54]. When the US government sold its remaining shares in GM in 2013, the bailout had cost taxpayers around US$11 billion [101]. Workers were forced to make considerable sacrifices during the reorganisation of the ‘New GM’. In 2009, 21,000 jobs were cut; a wage freeze was put in place for remaining workers; a funding program for unemployed workers was abolished; and a ‘no-strike’ agreement was put in place for the following six years [54, 102].

Between 1986 and 2002, a 16-year period prior to the financial collapse of GM, the company spent nearly US$35 billion (constant 2021 USD) on share buybacks. As Lazonick and Hopkins (2015) explain, if GM had saved those funds and earned a relatively modest 2.5% on it, the company would have had approximately US$60 billion (constant 2021 USD) spare at the time of the GFC [54].

Between 2015 and 2018, GM spent US$12.1 billion on share buybacks, on top of US$9.9 billion on dividends (constant 2021 USD). During this period, the company’s aggregate annual net income was approximately US$26 billion (constant 2021 USD). The decision to proceed with this large share buyback program was reportedly the result of an ongoing battle with so-called shareholder ‘activists’ – in this instance, hedge funds pressuring for larger shareholder returns [54]. The hedge fund-backed leader of these ‘activists’, former Goldman Sachs banker Harry Wilson, had been part of a team of ‘Wall Street’ experts that the Obama administration hired to organise GM’s government bailout [54].

In 2018 and 2019, GM cut more than 18,000 further jobs as part of a major company restructure [103, 104]. This decision was reportedly made to save the company around US$6 billion a year, by 2020, to be invested in, *inter alia*, research and development for electric and driverless vehicles [105].

## Discussion

This study shows that aggregate share buyback expenditure by US-listed corporations increased substantially between 1982 and 2021. As was the case during the period just before the GFC, the practice was seen to account for around half of the total annual value of shareholder ‘returns’ made by US-listed corporations in a number of recent years. Between 2012 and 2021, we estimate that aggregate share buyback expenditure by US-listed corporations reached US$9.3 trillion. In 2021 alone, share buyback expenditure by US-listed corporations was nearly US$1.2 trillion. According to some estimations, this is an amount larger than the 2021 US welfare budget (approximately US$1.1 trillion), and more than six times larger than the aggregate expenditure (approximately US$180 billion) on government aid promoting economic development and welfare in ‘developing countries’ by official members of the Organisation for Economic and Co-operation Development’s Development Assistance Committee [106, 107]. Unlike social welfare and development assistance, however, money spent on share buybacks mostly flows ‘upwards’ to those in the wealthiest echelons of predominately high-income country societies, a pattern of distribution linked with a range of poor health outcomes and health inequalities [36–38, 41–45, 108].

Relatedly, our findings strongly suggest that US-listed corporations are jeopardising the public’s health and the environment by increasingly distributing profits towards their shareholders instead of towards meaningful, and in some cases critical, investments for society. Our quantitative analysis revealed the stark increase in spending by US-listed corporations since the 1980s on shareholder 'returns’ relative to both net income (the foundation for future long-term investment), as well as capital, research and development expenditure (a proxy for actual long-term investment). As previously highlighted, share buybacks were a major contributor to this trend.

The three illustrative examples expose some of the general and sector-specific consequences of such corporate short-termism. In the first example, Pfizer, BioNTech, and Moderna were seen to be prioritising the short-term financial interests of their shareholders, such as by charging governments well above production costs and opposing IP waivers, over seeking to improve global vaccine access and equity, such as by sharing their mostly publicly funded technologies. It has been estimated that more than one million lives could have been saved if COVID-19 vaccines had been more equitably shared with lower-income countries in 2021 [109, 110]. While the blame for global COVID-19 vaccine inequity cannot be solely placed upon Pfizer, BioNTech, and Moderna, they clearly played an exacerbating role by driving vaccine prices beyond the reach of many countries in their quest for enormous profits [109, 110].

In the second example, Exxon Mobil, Shell, Chevron, and BP – four of the world’s largest fossil fuel corporations – were shown to be prioritising the short-term financial interests of their shareholders over other investments, such as in renewable and lower-carbon energy solutions. Such strong focus on ‘returning’ profits to shareholders was particularly apparent in times of record corporate profits, due in part to a surge in global oil prices, despite recognition by the industry itself of the urgent need for critical solutions to address climate change [55, 111].

In our third illustrative example, it was noted that General Motors – a company that a received a lifeline from taxpayers in 2009 – spent around US$22 billion on shareholder returns (US12.1 billion via share buybacks) between 2015 and 2018, before cutting more than 18,000 jobs in 2018 and 2019 to reportedly save US$6 billion a year. The impact of this decision on the health and well-being of workers and their families would have likely been considerable. Following the closure of one of General Motors’ plants, for instance, it was reported that an affected worker told journalists that they were unsure as to how they were now going to feed their family, including their 11-month-old child [112].

While our analysis focuses on share buybacks, the practice itself should be recognised as a symptom, rather than the disease. In many contexts, publicly listed corporations operate under the powerful ‘maximising shareholder value’ ideology, which, since the 1970s, has reportedly emerged to become the dominant principle of corporate governance [26, 53]. The rise of this ideology is often traced to Milton Friedman’s ‘doctrine’ published in the New York Times in 1970, entitled ‘The Social Responsibility of Business Is to Increase Its Profits’ [30]. One of the most highly influential neoliberal thinkers, Friedman considered corporate social responsibility as a ‘fundamentally subversive doctrine’ grounded in socialist political principles, and argued that the only responsibility of business should be to increase its profits primarily for the benefit of its shareholders [30]. Despite the emergence of newer and typically voluntary corporate forms that allegedly allow corporate profits to be pursued with ‘social purpose’ [4, 113], Friedman’s doctrine continues to be deeply engrained into contemporary thinking, law, and policymaking [4, 26, 33, 114]. Fundamentally, the ‘maximising shareholder value’ ideology is arguably one of a group of ideologies that largely serve to legitimise many contemporary institutional and governance arrangements which facilitate the distribution of society’s wealth and income from the poor to the rich, with minimal regard to broader social and ecological consequences [115]. Along with, *inter alia*, regressive tax policy and weak enforcement, the privatisation of essential services, the global intellectual property rights regime, ‘Chicago school’ antitrust, and the subordination of national public health regulations to trade and investment agreements [115–117], the normalisation of large-scale share buybacks can perhaps be understood as just one part of this system.

Despite serving largely as ‘symptomatic relief’, measures to better regulate (open market) share buybacks would nevertheless likely positively impact on public health and health equity to some degree. Recently, some governments have decided to implement taxes on share buybacks to partly address some of their potentially harmful impacts. In 2022, for instance, the U.S. Senate passed a rather modest one-percent tax on share buybacks as part of the Inflation Reduction Act, with the Canadian government announcing a two-percent tax on share buybacks shortly afterwards [118, 119].

Perhaps the most comprehensive policy reform would be for ‘open-market’ share repurchases to be prohibited, as was the case in many jurisdictions prior to the 1980s and 1990s, or at least strictly regulated [32]. In contrast to Friedman’s doctrine, the normative and theoretical foundations for implementing bans or restrictions on share buybacks link with the contention that the state has a legal and moral responsibility to mandate greater corporate social responsibility, not least because business corporations owe their ‘right to govern’ and most of their powers to generate profits to state concessions [114, 120, 121]. Importantly, this line of thinking supports the argument that company or corporate law can and should play a key role in pushing for largescale economic transformations towards sustainability [4, 122]. While a complete ban on share buybacks might be politically unfeasible, some scholars and politicians (especially in the US) have instead advocated for strictly regulating the practice when a corporation meets certain conditions [32, 123]. These conditions could include when a corporation reaches a certain size in terms of revenue or number of employees; when a corporation has received government assistance; when a corporation has recently cut jobs, compensates its executives above a certain threshold, or pays its workers below a certain threshold; and/or when a corporation externalises a substantial amount of costs onto society [32, 123–125]. Notably, under this non-exhaustive list, all of the corporations examined in our three illustrative examples would be made subject to the regulation in question.

A strength of this study is that it sourced a large amount of quantitative data dating back to 1982, which were complemented with data from company documents and the grey literature, to illustrate potential examples of ways in which share buybacks may influence population health and health equity. To the best of our knowledge, this is first study to analyse share buybacks through a public health lens. This study has several important limitations. First, Compustat does not separate data on the different forms of share buybacks, such as the buying back of shares on the ‘open market’ compared to the buying back of preferred shares. While we attempted to take this into account, in many cases, we were only able to provide estimates of ‘open market’ share buyback expenditure. Relatedly, it was beyond the scope of this study to verify data sourced from Compustat, such as by comparing them with data from official company reports. Another important limitation of this study is that it only included corporations listed on US stock markets. Notwithstanding the fact that US stock markets hold nearly 60% of the total world equity market value [61], an important avenue for future research could be to examine trends in share buyback expenditure by corporations listed on stock exchanges in other jurisdictions. Among other things, such work might be well placed to explore the potential of different share buyback-related norms and regulations to protect and promote public health [29]. Furthermore, this study only included three purposively selected illustrative examples. While we felt this was sufficient for the purposes of this explorative study, there is enormous scope to build on this study. Future work, for instance, could use case study research to help identify ways to effectively challenge the institutional and governance arrangements that facilitate and reinforce corporate short-termism, to the detriment of the public’s health, in various contexts.

## Conclusion

Increasing share buyback expenditure, in absolute terms and relative to investment, likely shapes many important socio-ecological determinants of ill-health and inequity. In recent decades, US-listed corporations have spent many trillions of dollars on share buybacks, with expenditure reaching nearly US$1.2 trillion in 2021. This is money that has flowed mostly to the wealthy. It also represents finite resources that corporate decision-makers have chosen not to allocate towards long-term investments that have the potential to protect and promote the welfare of diverse stakeholders. More broadly, meaningful long-term investments are essential to the development and prosperity of entire economies, as well as to address some of the greatest global threats to public health we face today, such as climate and ecological breakdown and the inequitable distribution of essential vaccines and medicines. We argue, therefore, that much more must be done to regulate share buybacks – the epitome of the ‘financialization of the real economy’ [25] – as part of broader efforts to address the ways in which corporations negatively impact on population health and health equity.

## Data Availability

The quantitative data that support the findings of this study are available from Wharton Research Data Services but restrictions apply to the availability of these data, which were used under license for the current study.

## Abbreviations

APA: Advanced purchasing agreement
COVID-19: Coronavirus Disease 2019
GFC: Global Financial Crisis
GM: General Motors
UK: United Kingdom
US: United States
USD: United States dollar
